# Validation of the Postgraduate Hospital Educational Environment Measure (PHEEM) in a sample of 731 Greek residents

**DOI:** 10.1186/s13104-015-1720-9

**Published:** 2015-11-30

**Authors:** Persa Koutsogiannou, Ioannis D. K. Dimoliatis, Dimitris Mavridis, Stefanos Bellos, Vassilis Karathanos, Eleni Jelastopulu

**Affiliations:** Department of Public Health, School of Medicine, University of Patras, Patras, Greece; Medical Education Unit, Department of Hygiene and Epidemiology, University of Ioannina Medical School, Ioannina, Greece; Department of Primary Education, University of Ioannina, Ioannina, Greece

**Keywords:** PHEEM, Validation, Greece, Hospital, Education environment, Measure, Residents

## Abstract

**Background:**

The Greek version of the Postgraduate Hospital Educational Environment Measure (PHEEM) was evaluated to determine its psychometric properties, i.e., validity, internal consistency, sensitivity and responsiveness to be used for measuring the learning environment in Greek hospitals.

**Methods:**

The PHEEM was administered to Greek hospital residents. Internal consistency was measured using Cronbach’s alpha. Root Mean Square Error of Approximation (RMSEA) was used to evaluate the fit of Structural Equation Models. Content validity was addressed by the original study. Construct validity was tested using confirmatory (to test the set of underlying dimensions suggested by the original study) and exploratory (to explore the dimensions needed to explain the variability of the given answers) factor analysis using Varimax rotation. Convergent validity was calculated by Pearson’s correlation coefficient regarding the participant’s PHEEM score and participant’s overall satisfaction score of the added item “Overall, I am very satisfied with my specialization in this post”. Sensitivity was checked by comparing good versus poor aspects of the educational environment and by satisfied versus unsatisfied participants.

**Results:**

A total of 731 residents from 83 hospitals and 41 prefectures responded to the PHEEM. The original three-factor model didn’t fit better compared to one factor model that is accounting for 32 % of the variance. Cronbach’s α was 0.933 when assuming one-factor model. Using a three-factor model (autonomy, teaching, social support), Cronbach’s α were 0.815 (expected 0.830), 0.908 (0.839), 0.734 (0.793), respectively. The three-factor model gave an RMSEA value of 0.074 (90 % confidence interval 0.071, 0.076), suggesting a fair fit. Pearson’s correlation coefficient between total PHEEM and global satisfaction was 0.765. Mean question scores ranged from 19.0 (very poor) to 73.7 (very good), and mean participant scores from 5.5 (very unsatisfied) to 96.5 (very satisfied).

**Conclusions:**

The Greek version of PHEEM is a valid, reliable, and sensitive instrument measuring the educational environment among junior doctors in Greek hospitals and it can be used for evidence-based SWOT analysis and policy.

## Background

Besides providing health services to the public, one of the most important targets of the health system is to train physicians who will provide these services. There are differences concerning the way trainers and trainees understand the perfect training environment [1, 2]. In addition, a major concern is the fact that training differs significantly not only between health systems of different countries due to cultural variations [3–5], but also between hospitals in the same national Health system if not even among departments of the same hospital.

It is of great importance to evaluate the quality of provided training in order to take corrective measures towards training improvement and to use failure events to improve work process [6]. The existence of an instrument that evaluates the quality of training programs in every day clinical practice is a step toward training perfection [3]. Continuing efforts targeting training improvements led to development of many instruments, created and validated in different countries [7]. These instruments include procedures for undergraduate medical students, such as DREEM [8], and instruments for various medical specialties, such as, anesthesiology, ATEEM [9], surgery, STEEM/OREEM [10], and ambulatory service, ACLEEM [11]. Further, there exists a generic instrument for the assessment of the educational environment of all junior doctors in hospitals, the Postgraduate Hospital Educational Environment Measure (PHEEM) [26], developed and validated by the Centre of Medical Education of the University of Dundee, UK, and being used worldwide [12–24].

PHEEM has been already translated and linguistically validated in Greek [25] but has not been psychometrically validated, considering the structural and cultural differences that may exist in the Greek national health care system [26, 27]. The aim of this study was to validate the translated PHEEM in the learning environment for junior doctors in Greek hospitals.

## Methods

### Ethical approval

The study was approved by the Medical Board of the University of Patras, and carried out in compliance with the Helsinki Declaration.

### The instrument

The original English PHEEM instrument consists of 40 items, 36 positive and 4 negative statements on a scale from “strongly disagree” to “strongly agree”, scored 0–4 on a five-point Likert scale (after reversing the negative ones), grouped into three subscales for perceptions of role autonomy, teaching, and social support [9]. Its Greek translation [25] was used for this psychometric validation. The original open-ended “Comments” was replaced by two specific ones, “If you could change one thing in this position what would it be” and “What would you not change”, after Whittle et al. [28] . To assess the comprehensibility and the cultural relevance of the items, the Greek version of PHEEM was tested on a group of 8 physicians. Based on this cognitive debriefing, some questions had to be slightly rephrased.

### Data collection

The questionnaire was initially distributed from January 2011 until February 2012 in paper form directly by the researchers to a convenient sample of doctors in specialty training programs in a broad selection of hospital departments and health care centers in West Greece. In November 2012 the questionnaire went online on Google Drive platform, emailing as much as possible residents in Greece, based on the records and the email addresses at the prefectural medical associations. Interns were asked to indicate, regarding their current training situation, their agreement with the statements using six options (strongly disagree, disagree, rather disagree, rather agree, agree, strongly agree). The four negative statements were scored in reverse so that in all items the higher the score the better the environment. Information on gender, age, hospital, residency year and specialty were also included. The participation in the study was voluntary and anonymous.

### Data analysis

Four instrument properties, namely reliability, validity, sensitivity (ability to detect differences between participants or groups of participants) and responsiveness (ability to detect changes when a participant improves or deteriorates) [29] should be tested. With this data we tested all except the last that was beyond the scope of this study. Mean scores for each item and domain and for the total instrument were calculated. For international comparison all scores are given in a 0–100 (%) scale, after converting the original 1- to 6-point scale, where 1 = 0, 2 = 20, 3 = 40, 4 = 60, 5 = 80 and 6 = 100 [30].

### Reliability

Cronbach’s alpha was used to measure internal consistency and unidimensionality of each set of items that refer to each of the factors [31]. Cronbach’s α higher than 0.7 shows acceptable (0.7–0.8), good (0.8–0.9) or excellent (>0.9) internal consistency; value >0.7 shows questionable (0.6–0.7), poor (0.5–0.6) or unacceptable (< 0.5) internal consistency [29]. Given the total scale alpha (α_scale_), expected subscale alphas were calculated with the Spearman–Brown formula, α_subscale_ = kα_scale_/(1 + (k−1)α_scale_), where k is the number of items of the subscale divided by the number of items of the total scale. Root Mean Square Error of Approximation (RMSEA) was used to evaluate the fit of Structural Equation Models; value of RMSEA smaller than 0.05 indicates very good fit, while values larger than 0.1 indicate poor fit, and intermediate values a fair fit.

### Validity

*Content* validity was addressed by the original study [9]. During the whole validation process, we were careful to spot any content issues that may arise. The same purpose was served by changing the open ended “Comments” by the two specific items described in “The instrument” paragraph.

*Construct validity* was tested with Confirmatory (CFA) and Exploratory (EFA) Factor Analysis, and with the underlying variable approach (UVA). CFA was used to test whether the set of underlying dimensions suggested by the original study [26] is adequate to explain all inter-relationships among the 40 observed ordinal items in the sample of Greek medical interns. EFA was performed to explore the dimensions needed to explain the variability of the answers; items with loadings <0.4 were excluded [21]. Statistical Package for Social Sciences (SPSS) Version 20 (SPSS Inc., Chicago, IL, USA) was used for these analyses. Further exploration was performed to test if some of the underlying dimensions are strongly correlated and could be represented by a single construct (i.e., one factor). Based on the idea that the observed ordinal variables are generated by a set of underlying continuous random variables, several methods have been developed to conduct factor analysis for ordinal data, using univariate (frequencies) and bivariate (crosstabs) information [32, 33]. Thus, we conducted factor analysis using the UVA in the statistical package LISREL [34], assessing items to factors as suggested by the original paper [26].

*Convergent validity* We calculated Pearson’s correlation coefficient between participant’s PHEEM score and participant’s overall satisfaction score of the added item “Overall, I am very satisfied with my specialization in this post”. In addition, the total PHEEM mean score was compared with the total satisfaction mean score, both statistically, accepting a *p* < 0.05 as significant difference, and educationally, accepting a 5 % difference as educationally minimum important difference (EMID), according to the quality of life field [35–37].

### Sensitivity

We are not aware of any educational differences among the Greek hospitals, in order to check the instrument’s ability to detect these differences. However, access to careers advice, counseling opportunities for failing residents, handbook for juniors etc. are not established in the educational environment in Greece; on the other hand, we expect no race or sex discrimination as well as good relation with colleagues; these expectations were tested comparing mean question scores. In addition, residents differ one from the other, and the same applies for teachers and posts, [1, 3–6]. Thus, it is reasonable to expect differences between the participants, ranging from very unsatisfied to very satisfied; this expectation was tested comparing the mean participant scores.

## Results

### Participants

We obtained 731 completed questionnaires (190 in paper and 541 online) from 55 % male and 45 % female interns, aged 24–49 (mean 33, standard deviation 3.5), being trained in 33 out of the 40 specialties, with a mean training time of 3.1 (1.5) years in total and 2.5 (1.2) in the current post, in 83 out of the 128 hospitals from 41 (80 %) of the 51 Greek prefectures.

### Reliability

Cronbach’s α was 0.933 when assuming one-factor model (internal consistency of the total questionnaire). When we used a three-factor model, using autonomy, teaching and social support as factors, Cronbach’s α were 0.815 (expected 0.830), 0.908 (0.839), 0.734 (793), respectively. Observed autonomy and social support alphas were slightly less than expected but within the same interpretative zone, while the observed teaching alpha was higher than expected and within one upper interpretative zone.

### Validity

#### Content validity

Soon after the electronic version was introduced, it was realized that there was a misconception with item seven (7) “*There is racism in this post”*. Several Greek residents interpreted this question as suggesting discrimination between the different specialties and not race discrimination; racism has gradually become a generic term in Greece meaning any discrimination. Therefore, the item 7 was further clarified to “*There is racism (race discrimination) in this post”*, a new item (41) “*There is a discrimination in this post against some specialties”* was added in the end of the questionnaire, and reliability and factor analyses were performed excluding the previous answers of the item seven. Furthermore, the two open-ended questions revealed important aspects that were not included in the original English version, such as easy and fast access to the internet, the way the specialty exams are carried out, and training in primary health care settings, in emergency settings, in outpatient and inpatient care.

#### Construct validity

We found very large correlations between the three factors: autonomy, teaching, social support (above 0.96 for all three pairwise combinations, data not shown) that suggest all three factors measure the same construct. The results using three-factor model and one-factor model are shown in Table 1. Loadings from the one-factor model are almost identical to the corresponding item loadings from the three-factor solution. The three-factor model gave an RMSEA value of 0.074 (90 % confidence interval 0.071, 0.076), suggesting a fair fit.

**Table 1:** Factor analysis (item loadings assuming three- and one-factor solutions, sorted by the one-factor total solution) and reliability (Cronbach’s alpha, last line) of our data

ID Item	OF	Three-factor solution			One-factor solution	
		A	T	S	Total	≥0.4
35 My clinical teachers have good mentoring skills	S			0.82	0.82	0.81
28 My clinical teachers have good teaching skills	T		0.79		0.79	0.78
02 My clinical teachers set clear expectations	T		0.74		0.73	0.74
06 Ι have good clinical supervision at all times	T		0.71		0.71	0.71
23 My clinical teachers are well organized	T		0.72		0.71	0.72
27 I have enough learning opportunities for my needs	T		0.69		0.69	0.69
15 My clinical teachers are enthusiastic	T		0.68		0.68	0.68
21 Access to my needs relevant educational programme	T		0.68		0.68	0.68
36 I get a lot of enjoyment out of my present job	S			0.69	0.68	0.68
30 Opportunities to acquire my grade practical procedures	A	0.68			0.67	0.69
22 I get regular feedback from seniors	T		0.67		0.66	0.67
34 Training makes me feel ready SpR/consultant	A	0.67			0.66	0.68
29 I feel part of a team working here	A	0.66			0.65	0.67
39 My clinical teachers provide me with good feedback	T		0.64		0.63	0.66
40 My clinical teachers promote mutual respect	A		0.64		0.63	0.69
10 My clinical teachers have good communication skills	T		0.61		0.60	0.60
14 There are clear clinical protocols in this post	A		0.59		0.59	0.62
03 I have protected educational time in this post	T		0.58		0.58	0.61
12 Ι am able to participate actively in educational events	T		0.59		0.58	0.59
31 My clinical teachers are accessible	T		0.58		0.58	0.58
18 I have the opportunity to provide continuity of care	A	0.56			0.56	0.58
33 Senior staff utilize learning opportunities effectively	T		0.52		0.53	0.54
04 I had an informative induction programme	A	0.52			0.52	0.52
38 Good counselling failing juniors	S			0.52	0.52	0.56
37 My teachers encourage me being independent learner	T		0.49		0.49	0.49
32 My workload in this job is fine	A	0.48			0.47	0.50
19 I have suitable access to careers advice	S			0.46	0.45	0.49
24 I feel physically safe within the hospital environment	S			0.46	0.45	0.44
05 I have the appropriate level of responsibility in this post	A	0.45			0.44	0.45
*08 I have to perform inappropriate tasks*	*A*	0.36			0.36	
16 I have good collaboration with doctors in my grade	S			0.36	0.35	
17 My hours conform to the ECC directives	A	0.35			0.35	
20 This hospital has good accommodation when on call	S			0.36	0.35	
25 There is a no-blame culture in this post	S			0.36	0.35	
01 Ι have a contract providing work hours information	A	0.34			0.33	
*13 There is sex discrimination in this post*	*S*			0.28	0.28	
*07 There is racism in this post*	*S*			0.27	0.27	
26 There are adequate catering facilities when on call	S			0.26	0.25	
09 There is an informative Junior Doctors Handbook	A	0.24			0.24	
*11 I am bleeped inappropriately*	*A*	0.20			0.19	
RMSEA^a^		0.074			0.090	0.097
Cronbach’s alpha (in parenthesis expected values)		0.815 (0.830)	0.908 (0.839)	0.734 (0.793)	0.933	

Negatively worded items are in italics, reverse scored so that its valence matches the positively worded items. Some items are slightly shortened

*OF* original factors, *A* autonomy, *T* teachers, *S* social support

^a^Corresponding 90 % confidence intervals are 0.071–0.076, 0.088–0.093, 0.095–0.099, respectively

**Fig. 1:**
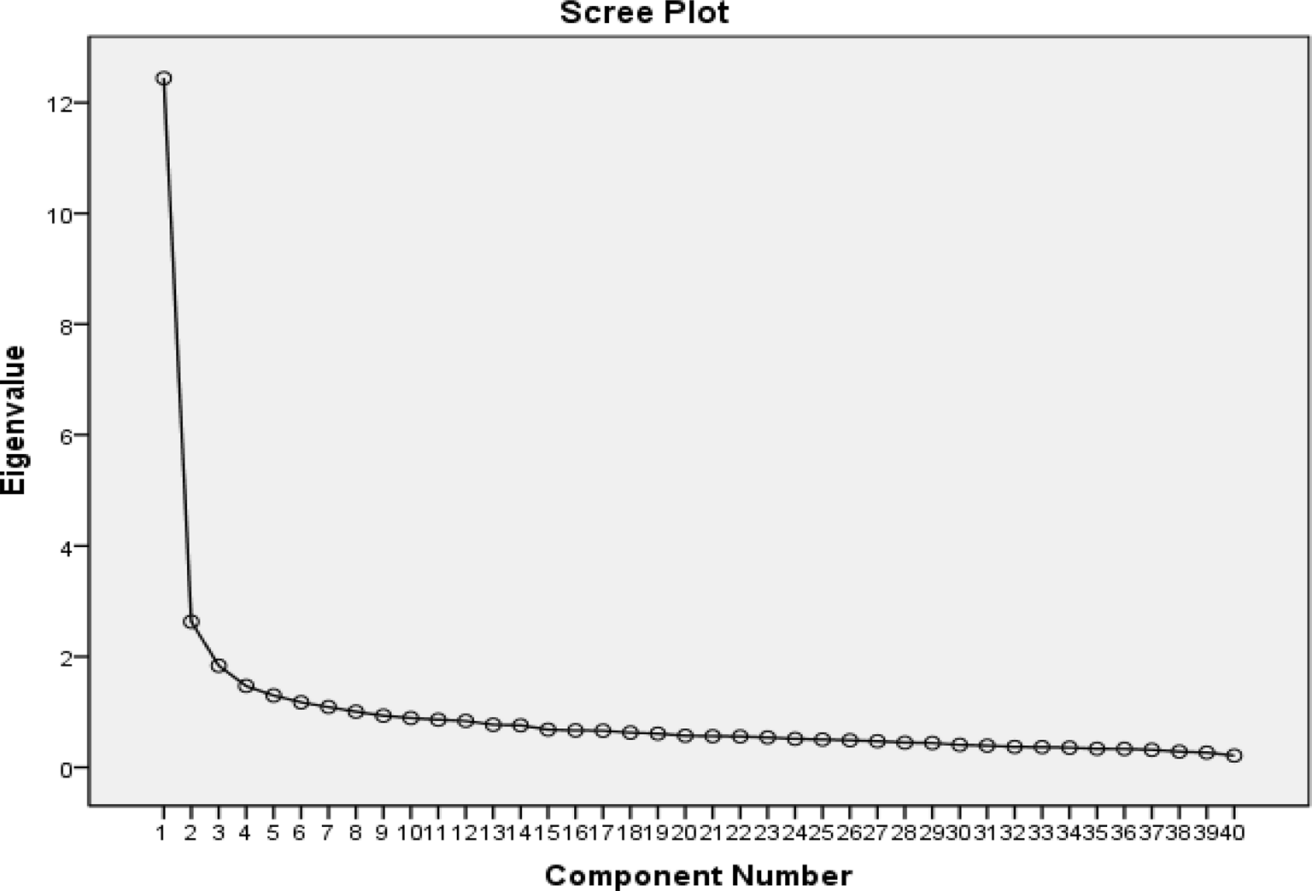
Scree plot of the factorial analysis and eigenvalues associated with principal components

Employing the one-factor analysis model in SPSS gave identical results, suggesting that the ordinal items have metric properties. More specifically the one-factor explained 32 % of the total variance, whereas at least 7 factors were needed to explain 50 % of the total variance. Using three factors explained 42 % of the total variance. However, it is very difficult to associate items to factors even after trying various rotation methods. The inflexion point in the scree plot is very subjective (Fig. 1). Using as criterion to keep all those factors with eigenvalue higher than 1.5 [21] yields three factors. Keeping all factors with an eigenvalue higher than 1.0, which is one of the default SPSS options, gives 8 factors. Using as criterion to keep only factors that increase the percentage of variance explained by at least 5 % [21], results in two factors. Excluding items 1, 7–9, 11, 13, 20, 25 and 26 that have loadings <0.4, the percentage of variance explained increased to 38 %.

#### Convergent validity

**Fig. 2:**
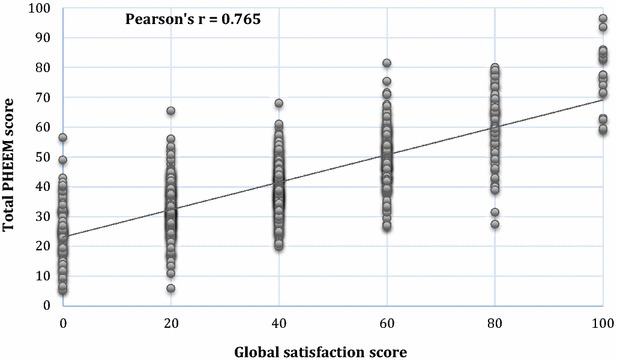
Correlation between participant global satisfaction and participant total PHEEM scores

Pearson’s correlation coefficient between participant total PHEEM score and participant overall satisfaction score was 0.765 (Fig. 2). The total PHEEM mean score (41.1 %) was statistically (*p* = 0.002) but not educationally (1.8 < 5 % = EMID) higher than the overall satisfaction mean score (39.3 %; Fig. 2, bottom).

### Sensitivity

**Fig. 3:**
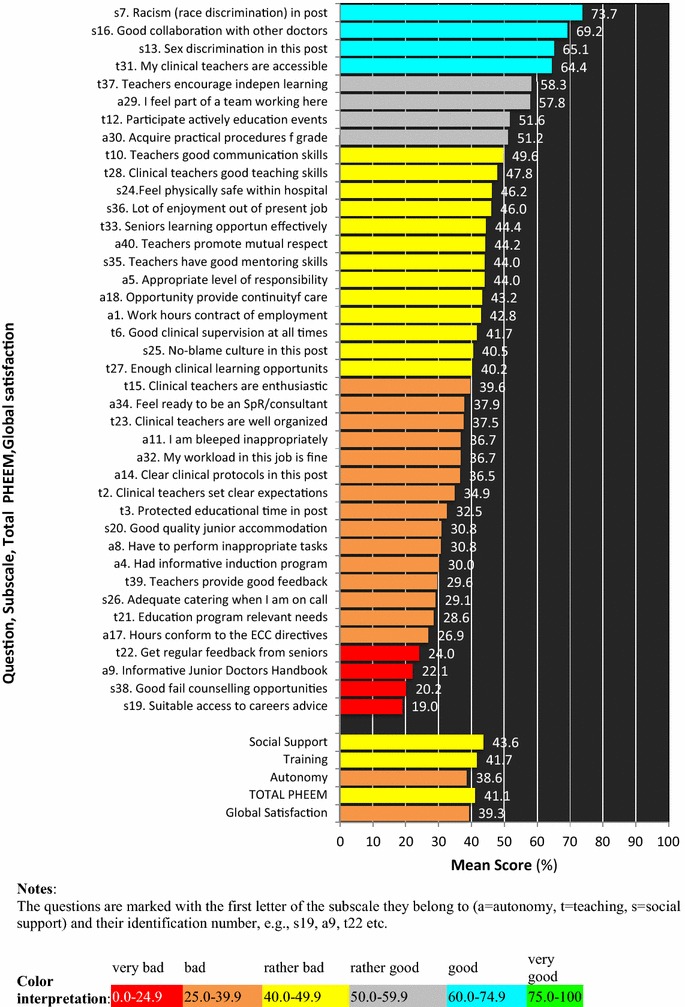
Mean score of every single item, of the three subscales, of the total PHEEM and of global satisfaction. The questions are marked with the first letter of the subscale they belong to (*a* autonomy, *t* teaching, *s* social support) and their identification number, e.g., s19, a9, t22 etc

**Fig. 4:**
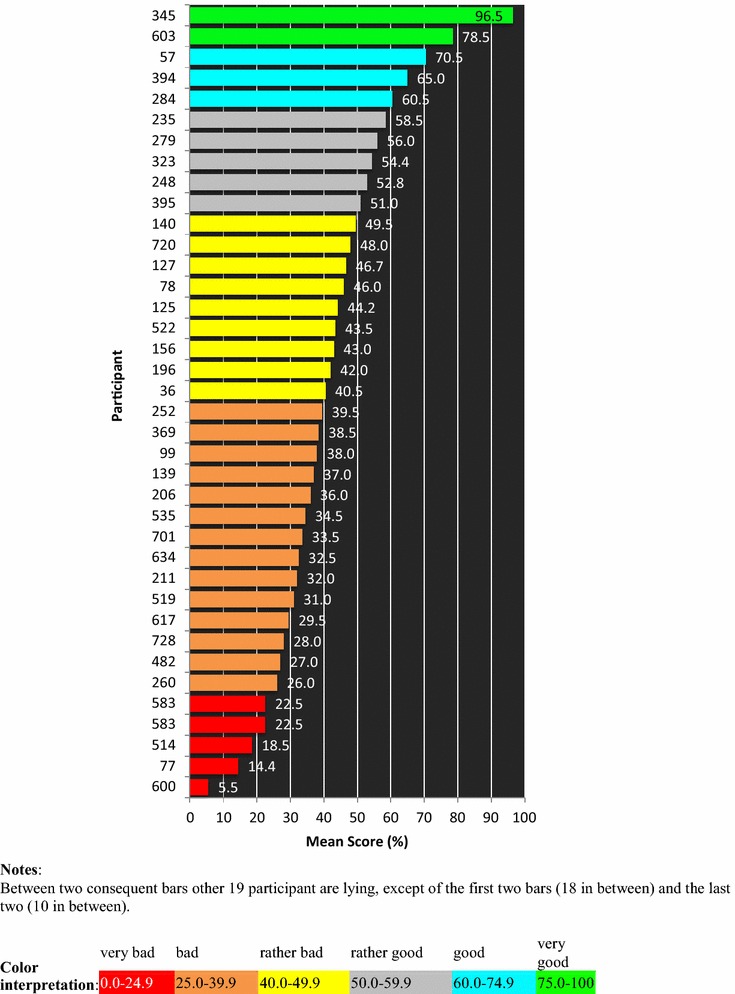
Participant mean score, presenting the first participant with the highest score (participant with ID 345) and the last with the lowest score (ID 600) and every twentieth intermediate participant. Between two consequent *bars* other 19 participant are lying, except of the first two *bars* (18 in between) and the last two (10 in between)

Mean question scores varied from 19.0 to 73.7 % (Fig. 3), while mean subscale scores fluctuated much less (autonomy 38.6 %, teaching 41.7 %, social support 43.6 %). Mean participant scores (Fig. 4) varied from 5.5 % (very unsatisfied) to 96.5 % (very satisfied).

## Discussion

The aim of this study was to validate the Greek version of PHEEM questionnaire in the working environment of the Greek National Health System hospitals, including participants from a wide range of hospitals and medical specialties. Validation of instruments is the process of determining whether there are grounds for believing that the instrument measures what it is intended to measure, and that it is useful for its intended purpose, by testing instrument’s reliability, validity, sensitivity and responsiveness [29].

### Reliability

Cronbach’s alpha of the total tool was higher than 0.90, indicating excellent internal consistency [27, 29]. This is very similar to the value of 0.91 [26]; 0.921 [2], 0.93 [12] and 0.92 [24]. However, Tavakol and Dennick [38] argue that a value of α > 0.90 may suggest redundancies and show that the test length should be shortened. There are at least three reasons for such high alphas: the correlation of the items on the scale (actual reliability), the length of the scale (the number of questions), and the length of the Likert scale (the number of response options). Response options, five in the original PHEEM, are six in this study and this adds to reliability [27]. The length of the scale might be an issue (see validity below). In any case, if we accept that other than actual reliability factors cause a 5 % or even 15 % increase in Cronbach’s alpha, it would still remain >0.80, indicating a very good reliability. Thus, we can conclude that our effort produced a reliable questionnaire.

### Validity

#### Construct validity

We used two different factor analysis models. Firstly we used factor analysis assuming the three subscales that were originally identified as autonomy, teaching, and social support [21]. Secondly, we used factor analysis assuming only one subsequent factor. Our results show that the loadings of each item didn’t vary significantly across the two models. We also found that treating ordinal items as continuous did not have an effect on the magnitude of the loadings. The three-factor model didn’t fit better compared to the one-factor model, meaning that categorizing questions into three independent factors is unnecessary for assessing the Greek specialty training environment. The one-factor model is in concordance with Boor et al. [14], but differs from Clapham et al. [2] who suggest 10 factors.

#### Content validity

Based on the factor analysis, the following five questions with loadings <0.3 should be removed (Table 1): Bleeped inappropriately; junior handbook; catering facilities; racism; sex discrimination (six more items should be removed if the loading cut point was 0.4). However, the expert panel consensus (consisted by 3 consultants, PK, EJ, ID, and 2 residents, VK, SB) decided that these questions are important aspects of the specialty training, thus they were not removed from the tool. In addition, especially for the Greek culture, we split the racism into two items “*There is racism (race discrimination) in this post”* and “*There is a discrimination in this post against some specialties”* (the last question’s loading was 0.68, while the loadings of the other questions remained unchanged). Furthermore, the two open-ended questions revealed important aspects that were not included in the original English version, such as “I have easy and fast access to the internet at my workplace”, “I am satisfied with the way the specialty exams are carried out”, “My training in primary healthcare settings is sufficient”, “My training in emergency inside and outside the hospital is sufficient”, “My training in outpatient care is sufficient”, and “My training in inpatient care within the wards is sufficient”. These were incorporated in the final Greek version (Appendix). However, we think the time has come for a meeting of the original version constructors with all worldwide translators, validators and users, to discuss and conclude for a new PHEEM version. The things are subject to constant change, the world changes, and perception too. In order the PHEEM to remain alive, it should also change. Exactly as cars, computers, word processors and other do. The meanwhile accumulated experience should be incorporated into a new version, the PHEEM v.1.

#### Convergent validity

The high correlation (in the upper quartile of Pearson’s correlation coefficient) between participant total PHEEM score and participant overall satisfaction score is consistent with the tool’s convergent validity. The same conclusion could be driven from the no educationally important difference of the two total means, while their statistical difference might be due to the very large sample size. Thus, there is no evidence that the Greek PHEEM is not a convergent tool. The illustrated convergent validity (a question on overall satisfaction) may rather subsume the items of the entire questionnaire, but the PHEEM has the ability to reveal where exactly the problem lies and how big it is. Thus, overall satisfaction cannot substitute the PHEEM instrument, but then the PHEEM can.

## Sensitivity and responsiveness

The low scores in items “access to careers advice”, “counseling opportunities for residents who fail”, “handbook for juniors”, and the high scores in “race or sex discrimination” and “good relation with colleagues” are as expected in Greece; i.e., the tool was sensitive enough to catch an existing situation. The same happened with the mean participant scores, varying from very unsatisfied till very satisfied; people differ among each other and the post environments are expected to differ as well; the tool was very sensitive to uncover this difference. Thus, we can conclude that the Greek PHEEM is a sensitive tool. Checking responsiveness was beyond the scope of this study. However, since sensitivity is a prerequisite for responsiveness [29], we can reasonably expect that the Greek PHEEM is also a responsive tool.

### Limitations

Using paper and electronic questionnaires might be a limitation; however, we calculated separate scores and we found no difference (39.6 vs. 41.7 %). Direct conclusions on responsiveness remain for a future work. Also, this instrument collects one stakeholder’s views, those of the trainees; perceptions of others—trainers, nurses, administrators, insurance, and of course patients—are missing; we need at least those of the two main players [39, 40]. Finally, we would like to emphasize—and this is a warning rather than a limitation—that this study focused on the validation of an instrument, not on measuring educational environment in Greek hospitals; thus, though they are based on a large sample including almost all medical specialties in university, tertiary and regional hospitals, results presented here, being a probably good estimation of this environment, should be interpreted with caution (the sample is not statistically representative).

### Conclusion

Through the validation process described above, there are grounds for believing that the Greek version of PHEEM measures what it is intended to measure, i.e., the education environment of the Greek hospitals, and that it is useful for its intended purpose. Even if the illustrated convergent validity (assessed by the single item of “global satisfaction”) may rather summarize all items of the PHEEM measure, the PHEEM has the ability to reveal where exactly the problem lies (e.g. “no suitable access to career advice” or “lack of regular feedback from seniors” etc.) and how big it is. Thus, we recommend to use the Greek version of PHEEM to monitor the educational environment and quality of hospital training in Greece and to assess and follow up on the effectiveness of potential corrective measures. A meeting of constructors, translators, validators and users could agree in a new version (v.1) of PHEEM.
